# A Novel Kindred with Familial Gastrointestinal Stromal Tumors Caused by a Rare *KIT* Germline Mutation (N655K): Clinico-Pathological Presentation and TKI Sensitivity

**DOI:** 10.3390/jpm10040234

**Published:** 2020-11-17

**Authors:** Mara Fornasarig, Daniela Gasparotto, Luisa Foltran, Michele Campigotto, Sara Lombardi, Elisa Del Savio, Angela Buonadonna, Fabio Puglisi, Sandro Sulfaro, Vincenzo Canzonieri, Renato Cannizzaro, Roberta Maestro

**Affiliations:** 1 Unit of Oncological Gastroenterology, Centro di Riferimento Oncologico di Aviano (CRO Aviano), IRCCS, 33081 Aviano, Italy; mfornasarig@cro.it (M.F.); rcannizzaro@cro.it (R.C.); 2 Unit of Oncogenetics and Functional Oncogenomics, Centro di Riferimento Oncologico di Aviano (CRO Aviano), IRCCS, 33081 Aviano, Italy; dgasparotto@cro.it (D.G.); os1@cro.it (S.L.); elisa.delsavio@cro.it (E.D.S.); 3 Unit of Medical Oncology and Cancer Prevention, Centro di Riferimento Oncologico di Aviano (CRO Aviano), IRCCS, 33081 Aviano, Italy; luisa.foltran@cro.it (L.F.); abuonadonna@cro.it (A.B.); fabio.puglisi@cro.it (F.P.); 4 Department of Medical, Surgical and Health Sciences, University of Trieste, 34127 Trieste, Italy; campigottomichele@gmail.com (M.C.); vcanzonieri@cro.it (V.C.); 5 Department of Medicine, University of Udine, 3310 Udine, Italy; 6 Unit of Pathology, Santa Maria Degli Angeli General Hospital, 33170 Pordenone, Italy; sandro.sulfaro@asfo.sanita.fvg.it; 7 Unit of Pathology, Centro di Riferimento Oncologico di Aviano (CRO Aviano), IRCCS, 33081 Aviano, Italy

**Keywords:** GIST, gastrointestinal stromal tumors, familial GIST, *KIT*, germline mutation

## Abstract

Gastrointestinal stromal tumors (GISTs), the most common mesenchymal tumors of the gastrointestinal tract, are characterized by activating mutations in *KIT* or *PDGFRA* genes. The vast majority of GISTs are sporadic, but rare hereditary forms have been reported, often featuring multifocality and younger age of onset. We here report the identification of a novel kindred affected by familial GIST caused by a *KIT* germline mutation in exon 13 (N655K). No family affected by hereditary GIST due to this *KIT* variant has been reported in literature so far. We were able to track the mutation in three members of the family (proband, mother, and second-degree cousin), all affected by multiple GISTs. Due to its rarity, the N655K variant is poorly characterized. We conducted in vitro drug sensitivity assays that indicated that most tyrosine kinase inhibitors (TKIs) currently included in the therapeutic armamentarium for GISTs have a limited inhibitory activity toward this mutation. However, when compared to a classical imatinib-resistant *KIT* mutation (T670I), N655K was slightly more sensitive to imatinib, and encouraging responses were observed with last-generation TKIs.

## 1. Introduction

Gastrointestinal stromal tumors (GISTs) are the most common mesenchymal tumors of the gastrointestinal tract and are thought to arise from the interstitial cells of Cajal (ICCs) [1,2]. The majority of GISTs (about 75%) harbor activating mutations in the *KIT* gene, most commonly in exon 11 and 9. Mutations in *PDGFRA* are detected in about 10–15% of GISTs. The fraction of GISTs devoid of *KIT* or *PDGFRA* mutations (~15%) is driven by rarer genetic alterations [1,2,3,4].

GISTs are essentially sporadic tumors, but rare familial forms associated with germline GIST-predisposing mutations have been reported. Among these are inactivating alterations of the *NF1* gene, responsible for type I neurofibromatosis, or defects in the genes encoding the succinate dehydrogenase complex (SDH), typically associated with the Carney–Stratakis GIST/paraganglioma syndrome [2,4,5,6].

Hereditary GISTs due to *KIT* or *PDGFRA* germline mutations are extremely rare, with very few cases reported so far. Tumor location and histology are similar to sporadic forms, but hereditary GISTs are often of early onset, multifocal, and associated with ICC hyperplasia. Other clinical manifestations include dysphagia, cutaneous hyperpigmentation, increased numbers of nevi, and mast-cell disorders [2,5,6].

We here report a case of a familial GIST syndrome due to a *KIT* exon 13 N655K germline mutation. The sensitivity of this rare *KIT* mutation to tyrosine kinase inhibitors (TKIs) currently approved or under trial for GISTs treatment was evaluated in vitro.

## 2. Materials and Methods

### 2.1. Histology and Immunohistochemistry

Formalin-fixed paraffin embedded (FFPE) surgical specimens were stained with hematoxylin and eosin (H&E). Immunohistochemical staining was performed by an automated immunostainer (Ventana, Roche, Basel, Switzerland) using the following antibodies: KIT/CD117 (polyclonal; 1:200; DAKO, Agilent, Santa Clara, CA, USA), CD34 (QBEnd 10, 1:1; DAKO), S100 protein (polyclonal; 1:2000; DAKO), alpha-smooth muscle actin (SMA) (1A4; 1:400; DAKO), Ki-67 (MIB-1; 1:200; DAKO), DOG1 (SP31; 1:100; Cell Marque, Merck, Darmstadt, Germany), and desmin (DE-R-11, 1:100; DAKO).

### 2.2. Molecular Analysis

DNA was extracted from FFPE tumor specimens by QIAamp DNA FFPE Tissue Kit (QIAGEN, Hilden, Germany). DNA from peripheral blood mononuclear cells was extracted with an EZ1 biorobot (QIAGEN).

Next generation sequencing (NGS) libraries were prepared with a TruSeq Custom Amplicon Low-Input kit (Illumina, San Diego, CA, USA) targeting the coding sequence of the *KIT, PDGFRA, BRAF, SDHA, SDHB, SDHC, SDHD, HRAS, KRAS, NRAS, SPRED1, NF1, NF2*, and *TP53*, as described [4]. Libraries were sequenced on an MiSeq platform (Illumina) using a v3 kit, 2 × 150 cycles. Data were analyzed with the Miseq Reporter software (v2.6.2), using the custom amplicon workflow and somatic variant caller. Mean amplicon coverage was 3200. Variants were analyzed with the VariantStudio 3.0 software (Illumina) and filtered by coverage > 50 and frequency ≥ 20%. Mutations detected by NGS were validated by Sanger sequencing on an ABI PRISM 3100 Genetic Analyzer (Thermo Fisher Scientific, Waltham, MA, USA).

### 2.3. Engineering of Ba/F3 Cells for the Expression of KIT Mutants

The murine interleukin-3 (IL-3) dependent Ba/F3 cell line is a cell model widely used in kinase studies. In fact, when ectopically expressed, certain tyrosine kinases can relieve Ba/F3 cells from IL-3 dependency, while tyrosine kinase inhibitors antagonize this effect [7,8].

Ba/F3 cells (ATCC, Manassas, VA, USA) were cultured in RPMI medium supplemented with murine interleukin-3 (IL-3; 10 ng/mL; PeproTech, Rocky Hill, IL, USA), 10% fetal bovine serum (FBS), and gentamicin (8 µg/mL).

Human *KIT* wild-type cDNA was cloned into the pLVX IRES ZsGreen viral vector (Clontech, Mountain View, CA, USA) and *KIT* exon 11 (W557_K558del), exon 13 (N655K), and exon 14 (T670I) mutant alleles were generated by PCR. Correct mutation introduction was checked by sequencing. To generate *KIT* lentiviral particles, human embryonic kidney HEK 293T cells (ATCC) were transfected with vectors encoding *KIT* mutants. HEK 293T cells were cultured in DMEM medium supplemented with 10% FBS and gentamicin (8 µg/mL). Ba/F3 cells were infected with HEK 293T-derived lentiviral supernatants in the presence of polybrene (8 µg/mL). Ectopically *KIT* expressing cells were selected by IL-3 withdrawal. Lentiviral infection efficiency was monitored in situ by green fluorescence. Expression of the ectopic *KIT* alleles was checked by Western blot. Briefly, cells were lysed in RIPA buffer (Santa Cruz Biotechnology, Dallas, TX, USA). Protein lysates were separated by SDS-PAGE and transferred onto a nitrocellulose membrane (Protran Whatman, Merck, Darmstadt, Germany). Expression analyses were performed with the following antibodies: anti c-KIT (H300; Santa Cruz Biotechnology) and anti-GAPDH (MAB374, Chemicon International, Temecula, CA, USA), for total protein loading normalization. A ChemiDoc imaging system (Bio-Rad, Hercules, CA, USA) was used for visualization. Ba/F3 cells expressing comparable levels of ectopic *KIT* mutants were used in TKI cell viability assays.

### 2.4. TKI Cell Viability Assay

Ba/F3 cells engineered to express the test mutation (*KIT* exon 13 N655K), an imatinib-sensitive mutation (*KIT* exon 11 W557_K558del), or an imatinib-resistant mutation (*KIT* exon 14 T670I) were seeded on a 48-well plate at a density of 7500 cells/mL. The following day, cells were exposed to the indicated doses of TKIs for 72 h, as previously described [7]. TKIs (Selleckchem, Munich, Germany) were dissolved in DMSO (dimethyl sulfoxide) as a vehicle. Drug dosage ranges were selected based on literature data and were as follows: imatinib (0–1000 nM), sunitinb (0–36 nM), regorafenib (0–480 nM), cabozantinib (0–24 nM), avapritinib (0–250 nM), and ripretinib (0–80 nM). Cell viability was evaluated by Trypan Blue staining and expressed as percentage of cells surviving the treatment, normalized to vehicle (DMSO)-treated cells. Four replicates per dose were evaluated. Results were confirmed on at least two independent Ba/F3 cell infections.

### 2.5. Ethical Approval

All procedures followed were in accordance with the Helsinki Declaration and with institutional and national ethical standards. The study was approved by the CRO Aviano institutional review board (IRB-04-2017). Written informed consent to be included in the study was obtained from patients.

## 3. Results

In March 2019, a 52-year-old woman was referred for genetic counseling after a diagnosis of multifocal GIST. She displayed diffuse freckles on her arms, legs, and trunk, and an atypical junctional nevus was surgically removed in February 2019. She had a history of estrogen-receptor-positive intraductal breast carcinoma diagnosed at the age of 43. She was also under surveillance for a thyroid nodule.

**Figure 1:**
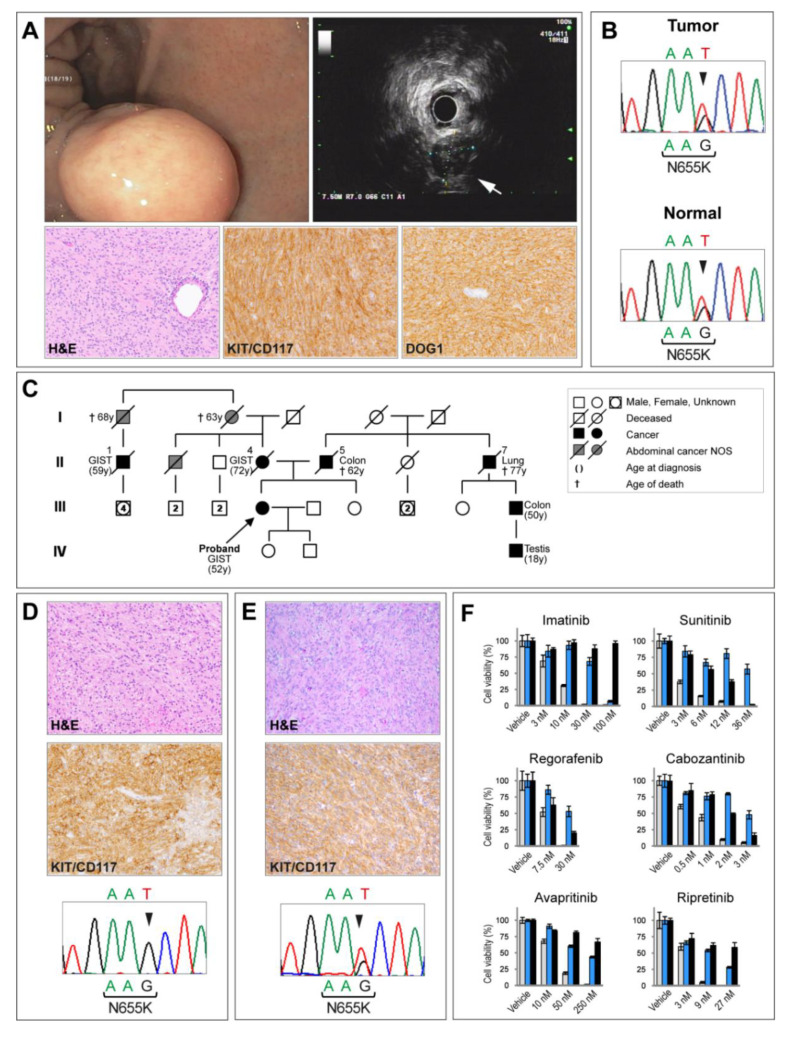
(**A**) Index case: endoscopic and histopathologic characteristics. Endoscopic (upper panel, left) and endoscopic ultrasound (upper panel, right) appearance of the submucosal gastric tumor of the index case (arrow). Lower panels: hematoxylin and eosin (H&E) staining of the lesion revealed bundles of spindle cells arranged in fascicles. Tumor cells displayed positive immunoreactivity for KIT/CD117 and DOG1 (original magnification: ×20). (**B**) Index case: molecular analysis. Sanger sequencing of tumor DNA revealed a T-to-G transversion at codon 655 of *KIT* exon 13 resulting in an N655K amino acid change (upper panel). The same *KIT* mutation was subsequently detected in the peripheral blood DNA (normal), indicating a germline origin (lower panel). (**C**) Family pedigree. (**D**) Index case’s mother: pathological and molecular characterization of the gastrointestinal stromal tumor (GIST). H&E staining of the tumor showing a proliferation of spindle cells. Tumor cells were positive for KIT/CD117 expression (original magnification: ×20). Molecular analysis of tumor DNA revealed a *KIT* exon 13 N655K mutation. No significant signal for the wild-type nucleotide (T) was detected, indicating that the mutation in the tumor of the index case’s mother was homozygous/hemizygous. (**E**) Index case’s second-degree cousin. Pathological and molecular characterization of the GIST. H&E staining showing spindle-shaped tumor cells disposed in fascicles. KIT/CD117 immunostaining demonstrating diffuse and strong membranous and cytoplasmic reactivity (original magnification: ×20). Sanger sequencing of tumor DNA revealing a *KIT* exon 13 N655K mutation. (**F**) In vitro tyrosine kinase inhibitor (TKI) sensitivity assay. Ba/F3 cells, engineered to express *KIT* mutant alleles, were treated with different TKIs at the indicated dosages. Cell viability is expressed as percentage of cells surviving the treatment, normalized to vehicle (DMSO)-treated cells. Color coding for *KIT* mutants: Light grey: imatinib-sensitive *KIT* exon 11 W557_K558del; Blue: the *KIT* exon 13 N655K mutation detected in the familial GIST syndrome described here; Black: imatinib-resistant *KIT* exon 14 T670I. Error bars represent standard error of the mean.

GIST was an incidental finding during bilateral salpingo-oophorectomy for uterine myomas and ovarian cyst (November 2018). Multiple nodules, the largest of 6 cm, were detected at the small bowel. At histopathological examination, these nodules showed spindle cells arranged in fascicles, and interlacing bundles with eosinophilic cytoplasm and elongated nuclei. Immunohistochemistry showed positivity for KIT/CD117 and DOG1, weak/focal reactivity for SMA and CD34, negativity for desmin and S100 (Figure 1A). Mitotic index was low (1 per 50 high-power fields). Diagnosis was of GIST of intermediate risk of progression according to Miettinen [9]. The diagnostic workup included a 18FDG-PET (fluorine-18-fluorodeoxyglucose positron emission tomography) that showed two lesions at the small bowel and one at the gastric fundus. Upper gastrointestinal endoscopy and ultrasound endoscopy showed a 2 cm submucosal nodule at the gastric fundus (Figure 1A). A second surgery was performed in January 2019 for resection of the gastric tumor and multiple small bowel nodules (size range 0.5–2 cm). Pathological examination corroborated the diagnosis of multinodular GIST. Molecular analysis, performed using a comprehensive GIST-specific NGS panel, revealed in both 2018 and 2019 tumor specimens a heterozygous T-to-G transversion at codon 655 of *KIT* exon 13 (c.1965T > G) resulting in an N655K amino acid change (Figure 1B). Besides the N655K *KIT* mutation, no other pathogenic mutation in GIST driver genes (*PDGFRA, BRAF, SDHA, SDHB, SDHC, SDHD, HRAS, KRAS, NRAS, SPRED1, NF1, NF2*, and *TP53)* was found.

Regarding family history (Figure 1C), the index case has a healthy sister (47 years old) with freckles on the trunk. Their mother died in 2012 at the age of 74 of metastatic GIST. She presented with multifocal intestinal GIST (positive for KIT/CD117, DOG1, and CD34; negative for S100, SMA, and desmin) and hepatic metastasis at diagnosis. Sequencing of tumor DNA revealed the same *KIT* N655K mutation detected in the proband (Figure 1D).

In 2000, the maternal second-degree cousin of the index case (II-1 at pedigree) was diagnosed at the age of 59 with multiple gastric and small-bowel mesenchymal malignant neoplasms compatible with GIST. The patient received two lines of combination chemotherapy for sarcomas (epidoxorubicine plus ifosfamide first, followed by CYVADIC, a combination of cyclophosphamide, vincristine, doxorubicin, and dacarbazine). Five years later (January 2006), he was referred to our center where PET/CT scan showed multifocal abdominal and hepatic increased uptake. Histological revision of the primary tumor supported the diagnosis of KIT/CD117-positive, DOG1-positive, GIST (immunohistochemistry was negative for CD34, S100, SMA, and desmin). Numerous microscopic GIST-like lesions (microGISTs) were also identified alongside the intestinal wall. Molecular analysis revealed a *KIT* exon 13 N655K mutation (Figure 1E). He started imatinib (400 mg daily). The disease progressed slowly until January 2007 when he underwent surgery for multiple abdominal masses. The patient was shifted to sunitinib for 15 months and, on progression, a rechallenge with imatinib at 800 mg allowed a progression-free interval of 9 months. A last line of treatment with nilotinib did not provide benefit and was stopped after 2 months.

The index case’s uncle (mother’s brother) and the maternal grandmother died of abdominal malignancy of unknown site and pathology (NOS). Colon cancer appeared as a recurrent event in the proband’s paternal branch.

In light of multifocal presentation, family history, and the recurrence of the same rare *KIT* exon 13 mutation in the neoplasms of the family, genetic testing was proposed to the proband. Analysis of peripheral blood DNA highlighted the presence of the same *KIT* N655K mutation detected in the tumors thus supporting its germline origin (Figure 1B).

Due to the intermediate risk of recurrence and the fact that *KIT* exon 13-mutated sporadic GISTs are in general scarcely responsive to imatinib, after curative surgery, the index case was put on close surveillance with alternating CT scan and 18FDG-PET every six months. A papillary thyroid carcinoma, follicular variant, was diagnosed in January 2020.

Genetic counseling was considered for the proband’s first-degree relatives. The sister refused genetic testing. Thus, annual abdominal ultrasound and upper-gastrointestinal endoscopy were suggested. The proband’s children were under age 18 and were therefore considered not eligible for genetic testing.

In sporadic GISTs, the *KIT* exon 13 N655K detected in this family is very uncommon. Thus, it is poorly characterized [7,8]. To address its sensitivity to TKIs approved or under trial for GIST treatment, we performed in vitro cytotoxicity assays. Specifically, the response to imatinib, sunitinib, regorafenib, avapritinib, cabozantinib, and ripretinib of Ba/F3 cells engineered to express the N655K mutation was compared with that of cells expressing a prototypical imatinib-sensitive (*KIT* exon 11 W557_K558del) and an imatinib-resistant (*KIT* exon 14 T670I) mutation (Figure 1F). Compared to the canonical *KIT* exon 11 sensitizing mutation (W557_K558del), the N655K mutation displayed a lower sensitivity to all TKIs tested. Compared to the reference resistant mutation (*KIT* exon 14 T670I), N655K appeared less responsive to sunitinib, regorafenib, and cabozantinib; it was instead slightly more sensitive to imatinib, especially at higher dosages, in keeping with previous results [7]. In addition, last-generation TKIs such as avapritinib and ripretinib [10] demonstrated a certain degree of activity toward this mutation (Figure 1F).

## 4. Discussion

Very few cases of *KIT/PDGFRA*-associated familial GIST syndromes have been reported so far. A PubMed search retrieved 51 reports, 45 describing hereditary GISTs due to germline *KIT* mutations, most of which involving exon 11, and 6 reports of familial GIST associated with germline *PDGFRA* mutations (Table 1) [11,12,13,14,15,16,17,18,19,20,21,22,23,24,25,26,27,28,29,30,31,32,33,34,35,36,37,38,39,40,41,42,43,44,45,46,47,48,49,50,51,52,53,54,55,56,57,58].

Here, we report a new kindred with familial GISTs caused by a *KIT* exon 13 germline mutation (N655K). In sporadic GISTs, *KIT* exon 13 mutations, which affect the ATP-binding pocket of the kinase, are uncommon and usually arise as secondary/imatinib-resistance events [1,2]. Among *KIT* exon 13 mutations, N655K is extremely rare, accounting for less than 0.1% of all GIST-associated *KIT* variants recorded in the Catalogue of Somatic Mutations in Cancer (COSMIC) database. With the only exception of a single, undetailed entry in the ClinVar database, to the best of our knowledge, there are no literature reports implicating this mutation in hereditary GISTs. Owing to its rarity, the N655K variant is poorly characterized and scanty literature data exist about its sensitivity to TKIs [7,8].

Our in vitro assay results indicate that N655K conveys a limited sensitivity to most TKIs approved for the treatment of GISTs. However, compared to a canonical resistance mutation (T670I), N655K was slightly more sensitive to imatinib, especially at higher dosages, a finding that is somehow in line with the response observed in the proband’s second-degree cousin. Always with reference to the T670I resistance mutation, N655K appeared more sensitive also to avapritinib and ripretinib. These latter are new-generation TKIs that have been proven to be effective in controlling a wide range of *KIT* and *PDGFRA* mutations, including classical resistant mutations [10].

The limited number of kindreds reported so far prevents genotype–phenotype correlations and the presentation pattern of these tumor forms and the management of patients carrying GIST-predisposing gene mutations still need to be defined [2,5].

As far as the treatment is concerned, there is currently no evidence supporting a differential therapeutic approach for GISTs developing in a background of germline *KIT*/*PDGFRA* mutations compared to sporadic GISTs, although tumor multiplicity and, hence, surgery-requiring complications (e.g., intestinal hemorrhages and occlusions) are more likely to occur in the former.

Definitively, when a familial GIST syndrome is suspected, based on early age of onset, tumor multifocality, and family history, genotyping is highly recommended and, if positive, counseling and predictive genetic testing should be offered to all first-degree relatives. Moreover, it is worth bearing in mind that individuals affected by hereditary GISTs seem to have an increased risk for other tumor types [2,5]. Indeed, our index case also developed breast and papillary thyroid cancers.

In our opinion, GIST-predisposing-mutation carriers should undergo close surveillance for early detection of cancer. This is even more important when the genetic setting makes the tumor poorly amenable to pharmacological inhibition, as in the family presented here, and the earliest diagnosis of neoplastic growth would increase the chance of curative surgery. Unfortunately, no specific guidelines exist on how to manage these subjects. In particular, consensus recommendations for clinical surveillance are lacking and whether regular esophagogastroduodenoscopy (EGD) or 18FDG-PET examinations are useful options remain open questions.

In this context, single case reports such as the one described here are fundamental to build up a body of information that may lay down the ground for the development of evidence-based guidelines.

## 5. Conclusions

In this paper we report the identification and characterization of a new kindred affected by multiple GISTs due to a rare *KIT* germline N655K mutation. Very few cases of syndromic *KIT/PDGFRA*-associated familial GISTs have been described so far. Case studies such as ours may help in defining the presentation pattern of these tumor forms and contribute to the formulation of clinical practice guidelines. In addition, in vitro evaluation of drug sensitivity may provide a basis for treatment personalization.

## Figures and Tables

**Table 1:** Currently reported cases of *KIT*- or *PDGFRA*-associated familial Gastrointestinal Stromal Tumors (GISTs).

Gene	Exon	Mutation	No. of Kindreds	Main Clinical Features [Reference]
*KIT*	8	D419del	1	systemic mastocytosis, multiple GISTs, dysphagia [11]
	9	K509I	2	systemic mastocytosis, multiple GISTs [12]; achalasia; mastocytosis, multiple GISTs [13]
	11	Y533C	1	multiple GISTs [14]
	11	W557R	4	multiple GISTs, skin hyperpigmentation, dysphagia [15]; multiple gastrointestinal autonomic nerve tumor [16]; multiple GISTs; skin hyperpigmentation [17]; multiple GISTs [18]
	11	W557S	1	multiple GISTs; lentigines [19]
	11	W557L K558E	1	multiple GISTs, hereditary breast cancer [20]
	11	V559A	7	multiple GISTs; lentigines, cafe-au-lait macules [21]; multiple GIST, cutaneous hyperpigmentation [22]; multiple GISTs, melanosis, lentiginosis, hyperpigmentation, dysphagia [23]; multiple GISTs, hyperpigmentation, urticaria pigmentosa [24]; multiple GISTs, cutaneous hyperpigmentation [25]; multiple GISTs [26]
	11	V559_V560del	1	multiple GISTs, cutaneous hyperpigmentation [27]
	11	V560del	1	multiple GISTs [28]
	11	V560G	1	multiple GISTs, cutaneous hyperpigmentation [29]
	11	V560A	1	multiple GISTs [29]
	11	Q575_P577delinsH	1	rectal GIST [30]
	11	L576P	2	multiple GISTs; skin hyperpigmentation, achalasia-like stenosis [31]; multiple GISTs [32]
	11	L576_P577insQL	1	multiple GISTs, cutaneous hyperpigmentation [33]
	11	D579del	7	multiple GISTs, cutaneous hyperpigmentation, dysphagia [34]; GIST [35]; GIST, cutaneous hyperpigmentation [36]; multiple GISTs [37]; multiple GISTs [38]; multiple GIST, nevi, hyperpigmentation [39]
	13	K642T	1	multiple GISTs, dysphagia [40];
	13	K642E	7	multiple GISTs, breast cancer [41]; multiple GISTs, dysphagia, multiple nevi and lentigines [42]; multiple GISTs [43]; multiple GISTs including rectal GIST [44]; multiple GISTs, dysphagia, pigmentary defects (hyper- and hypopigmentation) [45]; multiple GISTs including rectal GIST, pigmentary defects (hyper- and hypopigmentation) [46]
	13	N655K	1	multiple GISTs, lentigines, atypical junctional nevus, breast and thyroid cancer [present report]
	17	D820Y	3	multiple GISTs, dysphagia [47]; multiple GISTs [48]; multiple GISTs including rectal GIST [49]
	17	D820G	1	multiple GISTs [50]
	17	N822Y	1	multiple GISTs [51]
*PDGFRA*	12	Y555C	1	multiple GISTs, intestinal neurofibromatosis, glaucoma, coarse facies, broad hands [52]
	12	V561D	1	multiple GISTs, fibrous tumors, lipoma [53,54]
	14	P653L	2	multiple GISTs, fibrous tumors, inflammatory fibroid polyps [55,56]
	18	D846Y	1	multiple GISTs, broad hands [57]
	18	D846V	1	multiple GISTs, coarse facies/skin, broad extremities [58]
